# A Bayesian spatio-temporal framework to assess the effect of seasonal malaria chemoprevention on children under 5 years in Cameroon from 2016 to 2021 using routine data

**DOI:** 10.1186/s12936-023-04677-1

**Published:** 2023-11-11

**Authors:** Arnold Fottsoh Fokam, Toussaint Rouamba, Sekou Samadoulougou, Yazoume Ye, Fati Kirakoya-Samadoulougou

**Affiliations:** 1 Innovations for Poverty Action, Cocody, Quartier Val Doyen, Abidjan, Côte d’Ivoire; 2 ICF, Maroua, Cameroon; 3 Clinical Research Unit of Nanoro, Institute for Research in Health Sciences, National Center for Scientific and Technological Research, 42, Avenue Kumda‑Yoore, BP 218 Ouagadougou CMS 11, Ouagadougou, Burkina Faso; 4 Evaluation Platform on Obesity Prevention, Quebec Heart and Lung Institute Research Center, Quebec City, QC G1V 4G5 Canada; 5 Centre for Research on Planning and Development, Laval University, Quebec, G1V 0A6 Canada; 6 ICF, 530 Gaither Road, Rockville, MD 20850 USA; 7 Centre de Recherche en Epidémiologie, Biostatistique et Recherche Clinique, Ecole de Santé Publique, Université Libre de Bruxelles, Route de Lennik, 808, Bruxelles, Brussels, 1070 Belgium

**Keywords:** Seasonal, Malaria, Chemoprevention, Temporal series, Bayesian analysis, Cameroon

## Abstract

**Background:**

Malaria affects millions of Cameroonian children under 5 years of age living in the North and Far North regions. These regions bear the greatest burden, particularly for children under 5 years of age. To reduce the burden of disease in these regions, Cameroon adopted the Seasonal Malaria Chemoprevention (SMC) in 2016 and has implemented it each year since its adoption. However, no previous studies have systematically assessed the effects of this intervention in Cameroon. It is important to understand its effect and whether its implementation could be improved. This study aimed to assess the effect of SMC in Cameroon during the period 2016–2021 on malaria morbidity in children under 5 years of age using routine data.

**Methods:**

Data on malaria cases were extracted from the Cameroon Health Monitoring Information System (HMIS) from January 1, 2011, to December 31, 2021. Health facilities report these data monthly on a single platform, the District Health Information System version 2 (DHIS2). Thus, a controlled interrupted time-series model in a Bayesian framework was used to evaluate the effects of the SMC on malaria morbidity.

**Results:**

SMC implementation was associated with a reduction in the incidence of uncomplicated malaria cases during the high-transmission periods from 2016 to 2021. Regarding the incidence of severe malaria during the high-transmission period, a reduction was found over the period 2016–2019. The highest reduction was registered during the second year of implementation in 2017:15% (95% Credible Interval, 10–19) of uncomplicated malaria cases and 51% (47–54) of confirmed severe malaria cases.

**Conclusion:**

The addition of SMC to the malaria intervention package in Cameroon decreased the incidence of uncomplicated and severe malaria among children under 5 years of age. Based on these findings, this study supports the wide implementation of SMC to reduce the malaria burden in Cameroon as well as the use of routine malaria data to monitor the efficiency of the strategy in a timely manner.

**Supplementary Information:**

The online version contains supplementary material available at 10.1186/s12936-023-04677-1.

## Background

The malaria burden remains high among children under 5 years of age in Cameroon, especially in the North and Far North regions [1]. In 2021, the World Health Organization (WHO) estimated that 6.9 million confirmed malaria cases and 14,841 malaria-related deaths would be registered in the country [2]. Two (North and Far North) of the country’s ten regions account for nearly 60% of national malaria deaths among children under 5 years [1]. In its 2014–2018 National Malaria Strategic Plan (reconducted in 2019–2023), Cameroon adopted the Seasonal Malaria Chemoprevention (SMC) strategy to reduce the burden of malaria among children under 5 years of age in the North and Far North regions, where malaria transmission is seasonal [1]. SMC consists of administering a maximum of four treatment courses of sulfadoxine–pyrimethamine plus amodiaquine at monthly intervals to children aged 3–59 months in areas with high seasonal malaria transmission [3].

From 2016 to 2021, between July and October, the SMC was implemented annually in all North and Far North health districts. This intervention was introduced in addition to the policy of free-of-charge malaria care for children under 5 years of age and other malaria prevention and treatment interventions. Despite 6 years of SMC implementation in Cameroon, the malaria burden remained high in both regions. Between 2016 and 2021, the overall trend of malaria morbidity among children under 5 years increased from 29 to 48% in the northern region and from 34 to 66% in the far northern region [2]. This raises concerns about the effectiveness of SMC in reducing malaria morbidity among children under 5 years in the two regions. Other countries have documented significant reductions in the malaria burden through rigorous studies, attributing it to the implementation of SMC [4]. Indeed, with improvements in malaria monitoring and evaluation systems, and substantial advances in computational tools, analysis, and visualization of routine data in public health, the effectiveness of interventions that use routine data has increased, especially in cases in which randomized controlled trials are not possible. However, no study has systematically assessed the effects of this intervention in Cameroon.

To address this gap, this study aims to assess the effects of SMC on malaria morbidity among children under 5 years in the North and Far North regions of Cameroon during the period 2016–2021 using routine data reported through the HMIS.

## Methods

### Study area and population

Cameroon is in Central Africa, bordered by the Atlantic Ocean and six other countries: Nigeria in the west, Chad in the north, the Central African Republic in the east, and Congo, Gabon, and Equatorial Guinea in the south. In 2022, the population of Cameroon is estimated at 27.8 million [5], with a surface area of 475,442 km^2^. The country has ten regions (Adamawa, Centre, East, Far North, Littoral, North, Northwest, South, Southwest, and West) and two metropolises (Yaoundé and Douala) subdivided into three main epidemiological facies linked to geo-climatic variations:

- Sudano-Sahelian facies (Far North and North regions).
- The great interior plateau savannah (Adamawa region).
- The great equatorial forest (all 7 regions of the south).

The existing climatic conditions favour the development of malaria vectors and parasites.

### Data sources

Data on attendance and malaria cases were extracted from the Cameroon HMIS from January 1, 2011, to December 31, 2021. Health facilities (public and private) report these data monthly on a single platform, the District Health Information System version 2 (DHIS2) [6], which facilitates data aggregation at the health area, health district, regional, and central levels. All data in the HMIS were disaggregated into three population groups: pregnant women, children under 5 years of age and, individuals over 5 years of age excluding pregnant women.

Two outcomes were considered: confirmed uncomplicated malaria and severe malaria. The following definitions were adopted: a confirmed malaria case is characterized by the presence of a positive malaria test (either a rapid diagnostic test or blood smear) in a symptomatic patient (fever, headache, or other malaria-related symptoms) [7]. On the other hand, an uncomplicated malaria case refers to a patient with symptomatic malarial parasitaemia without signs of severity or evidence of vital organ dysfunction confirmed by microscopy or rapid diagnostic test [6]. Severe malaria is defined as a confirmed case of malaria with signs of severe illness or evidence of vital organ dysfunction requiring hospitalization [8].

In this study, two age groups were used: children under 5 years as the intervention group and individuals 5 years and older, excluding pregnant women, as the comparison group. The incidence rates of uncomplicated and severe malaria per 1000 inhabitants for each group and individual health district were computed by dividing the number of confirmed cases by the total population specific to each group living in the health district.

### Statistical analytic model

A controlled interrupted time-series model was fitted separately on district-aggregated monthly uncomplicated and severe malaria cases reported in DHIS2 during 2011–2021 to assess the impact of the SMC on malaria morbidity in children under 5 years. Cameroon implemented SMC over a continuous period of 4 consecutive months each year; therefore, no level change (for an abrupt intervention effect) was assumed, but only a slope change. Moreover, after 4 consecutive months of implementation, the intervention was withdrawn for the next 8 months until its reintroduction in the following year. Therefore, no change in the level was assumed at withdrawal, and the remaining slope change at withdrawal was not null. Thus, the controlled interrupted time-series analysis accounted only for changes in trend (increase or decrease in slope) that followed the intervention [9] and its withdrawal.

Moreover, due to common exposure between neighbouring health districts and time points, and considering the characteristics of malaria vectors, a similar spatial correlation occurred in our routine data due to the populations’ exposure to environmental and climatic conditions at locations that are geographically close. To examine this geographical correlation, geostatistical models that consider spatial correlations were used. A Bayesian Conditional Autoregressive (CAR) approach was employed in this study to account for the spatial correlation in health district-level incidence and to smooth malaria rates, which helped highlight the spatial pattern of malaria burden and produce unbiased parameter estimates [10].

This allowed the introduction of health district-specific effects. A controlled interrupted time-series model was then constructed within a Bayesian geostatistical framework. This framework considers spatial components by introducing spatial correlations into the covariance matrix of random effects. As count data were used for confirmed uncomplicated and severe malaria cases, a negative binomial model was used to account for overdispersion. Finally, the Bayesian negative binomial model was adjusted for monthly, seasonal, and annual trends. Descriptive statistics were conducted using Stata (version 14) and controlled interrupted time-series regression analyses were performed in R software, version 4.2.2, using Integrated Nested Laplace Approximation package (R Foundation for Statistical Computing, Vienna, Austria). Details on the model formulations, metric measures of accuracy, marginal risk, and posterior marginal distribution of the parameters are provided in Additional files 1, 2, 3 and 4.

## Results

The North and Far North regions of Cameroon experienced an increase in the incidence of confirmed malaria cases among both children under and over 5 years from 2011 to 2021. Among children under 5 years of age, three phases, which followed global trends in the annual number of registered uncomplicated malaria cases in these regions, were observed. Before SMC implementation, there was a continuous annual increase, rising from 44,876 cases in 2011 to 138,002 in 2015 (Table 1). After the introduction of the SMC, there was a short period (2015–2016) of decline to 80,042 cases in 2016, before a continuous yearly increase to 259,328 cases in 2021. The incidence of uncomplicated malaria in children under 5 years during the study period (January 2011 to December 2021) was nearly three times higher than that in individuals aged 5 years and older.

**Table 1:** Trends of uncomplicated and severe malaria cases in the North and Far North regions

SMC intervention	Years	Groups	Suspected cases	Suspected cases tested	Testing rate	Uncomplicated malaria-confirmed cases	Severe malaria-confirmed cases	Uncomplicated malaria incidence (per 1000)	Severe malaria incidence (per 1000)
Before	2011	Comparison group	204,283	170,103	83.27	75,009	62,729	30.9	25.9
		Intervention group	156,163	88,652	56.77	44,876	34,335	81.9	62.7
	2012	Comparison group	209,102	211,641	100.00	86,201	71,616	35.0	29.1
		Intervention group	141,653	127,167	89.77	50,177	35,808	90.5	64.6
	2013	Comparison group	207,820	210,103	100.00	85,554	71,274	33.9	28.2
		Intervention group	144,226	130,927	90.78	51,562	36,847	90.5	64.7
	2014	Comparison group	116,664	113,659	97.42	49,685	45,406	18.6	16.9
		Intervention group	173,797	171,998	98.96	77,444	70,999	128.3	117.7
	2015	Comparison group	319,231	499,043	100.00	169,826	176,800	63.3	65.9
		Intervention group	260,656	339,910	100.00	138,002	120,426	228.0	198.9
After	2016	Comparison group	260,922	454,633	100.00	128,474	140,339	48.1	52.5
		Intervention group	170,362	249,148	100.00	80,042	68,815	132.8	114.2
	2017	Comparison group	552,405	600,364	100.00	173,619	191,613	60.9	67.2
		Intervention group	339,611	345,932	100.00	100,173	43,839	148.6	65.0
	2018	Comparison group	637,433	621,564	97.51	188,467	229,044	66.1	80.3
		Intervention group	409,832	381,857	93.17	119,585	87,201	177.2	129.2
	2019	Comparison group	786,047	740,623	94.22	259,815	220,480	88.2	74.8
		Intervention group	524,929	491,641	93.66	171,951	127,665	246.9	183.3
	2020	Comparison group	882,318	819,678	92.90	302,655	266,330	97.5	85.8
		Intervention group	568,087	534,179	94.03	203,222	166,532	271.7	222.6
	2021	Comparison group	102,4769	978,413	95.48	384,969	263,890	119.6	81.9
		Intervention group	692,150	653,581	94.43	259,328	192,999	340.4	253.3

### Trends of uncomplicated and severe malaria-confirmed cases in the North and Far North regions of Cameroon from 2011 to 2021

**Fig. 1:**
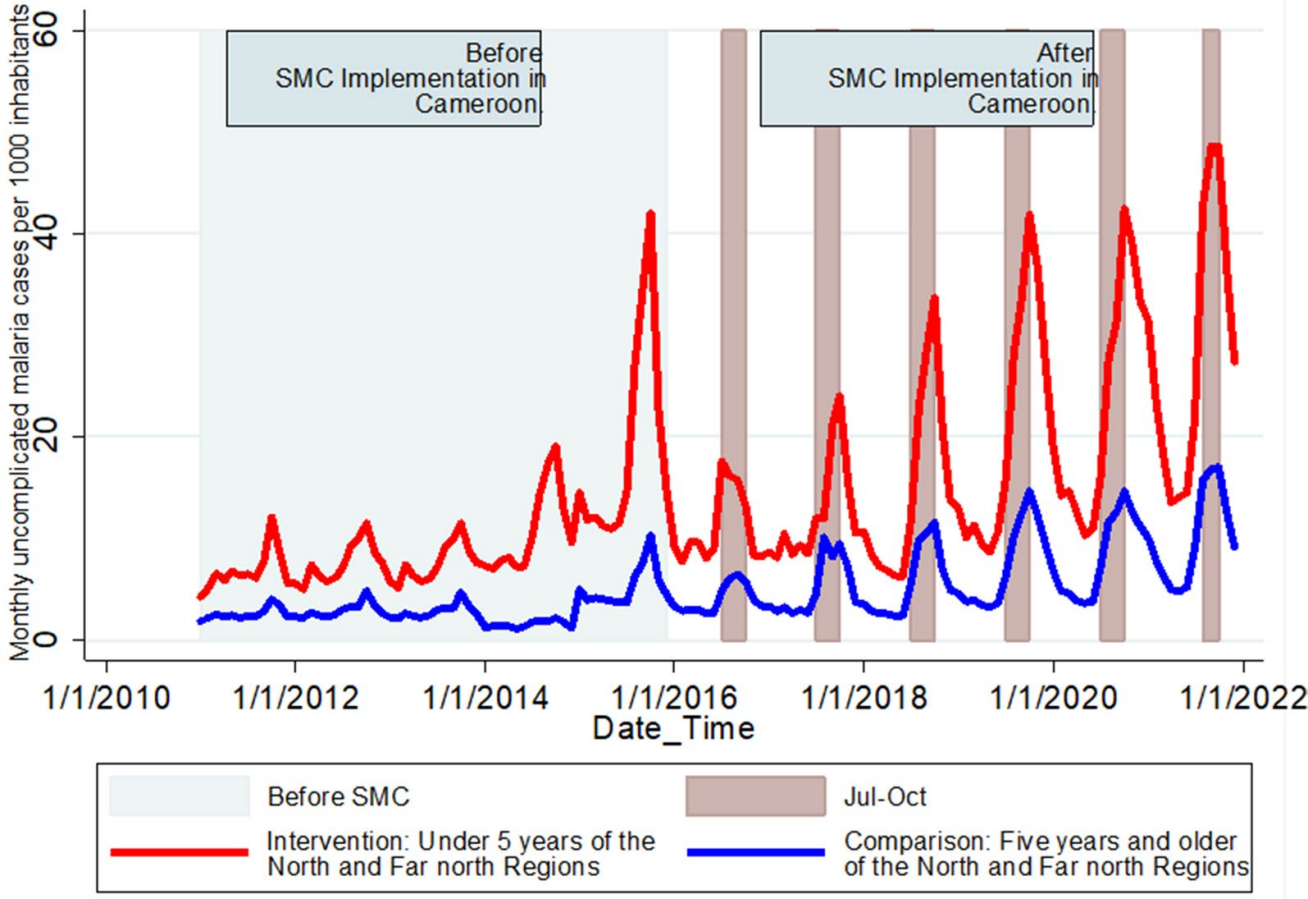
Monthly incidence of uncomplicated malaria over time by age group in North and Far North regions

The average monthly uncomplicated malaria incidence in children under 5 years increased steadily, from 81.9 cases per 1000 inhabitants in 2011 to 228.0 cases per 1000 inhabitants in 2015. This increase from 2011 to 2015 was followed by a decrease to 132.8 cases per 1000 inhabitants in 2016, subsequent to the first year of SMC implementation. However, after this 1-year decrease between 2015 and 2016, a second increase was registered from 2016 to 2021, reaching 340.4 cases per 1000 inhabitants in 2021. In the older age group, a steady increase in monthly incidence from 2011 to 2015 was followed by a decrease in 2016, and a second increase until 2021. Temporal trends in uncomplicated malaria incidence showed a strong annual seasonal pattern with one peak from July to November (Fig. 1).

**Fig. 2:**
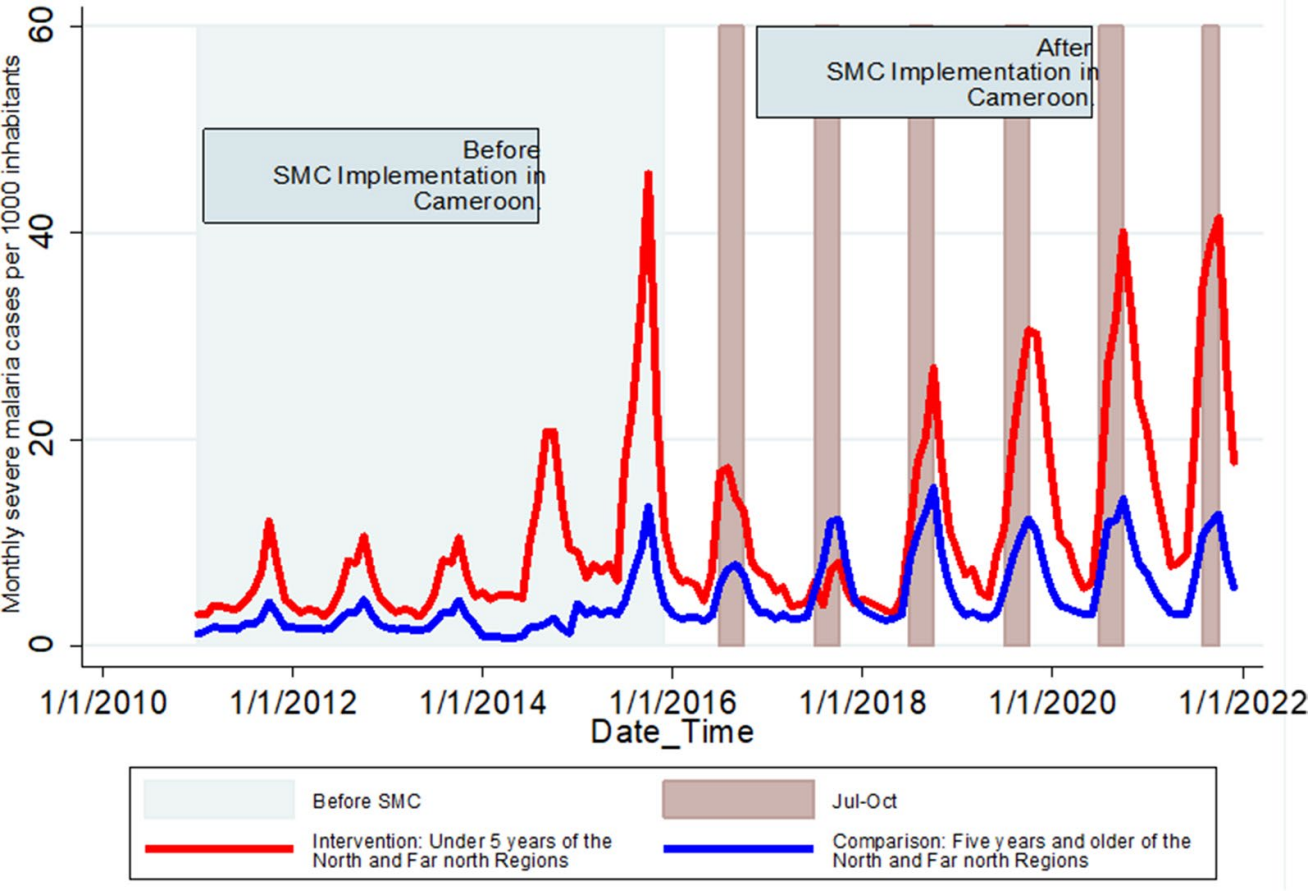
Monthly incidence of severe malaria over time by age group in North and Far North regions

For severe malaria also, the North and Far North regions in Cameroon experienced an increase in confirmed cases and incidence among children under 5 years and individuals over 5 years from 2011 to 2021. Among children under 5 years, three phases were observed in these regions that follow the global trends in the annual number of registered severe malaria cases. First, there was a continuous yearly increase from 34,335 to 2011 to 120,426 in 2015 (Table 1), followed by a 2-year period of decline to 43,839 in 2017, and a continuous yearly increase to 192,999 in 2021 (Table 1). Severe malaria incidence in children under 5 years during the study period (January 2011–December 2021) was around three times higher than that in individuals 5 years and older (Fig. 2). The average monthly incidence of severe malaria in children under 5 years increased steadily, from 62.7 cases per 1000 inhabitants in 2011 to 198.9 cases per 1000 inhabitants in 2015. In the older age group, a steady increase in the monthly incidence of severe malaria was observed from 2011 to 2015. However, this increase was followed by a 1-year decrease in 2016, in contrast to the 2-year decrease for children under 5 years, and then by a second yearly increase from 2017 to 2021. Similar to uncomplicated malaria, the temporal trends of severe malaria incidence showed a strong annual seasonal pattern, with one peak from July to November (Fig. 2).

#### Impact of SMC

Since the implementation of SMC in 2016, the estimates presented in Table 2 have consistently shown a positive association between uncomplicated malaria incidence and SMC in Cameroon. In 2016, a proportional reduction of 12% [Incidence Rate Ratio (IRR) = 0.88, 95% Credible Interval (CrI): 0.85–0.93] was observed. This reduction was followed by the greatest proportional reduction of 15% (IRR = 0.85, 95% CrI 0.81–0.90) in 2017. Subsequently a slight decrease of 10% was observed in 2018. However, the impact of SMC has stabilized at 10% with consistent reductions in uncomplicated malaria incidence during the last 3 years (2019, 2020, and 2021).

**Table 2:** Effects of SMC on malaria incidence estimated from the controlled interrupted time-series model

Periods	Uncomplicated malaria cases		Severe malaria cases	
	IRR	95% BCI	IRR	95% BCI
2011–2015	1		1	
2016	0.88*	0.84–0.93	0.90*	0.84–0.95
2017	0.85*	0.81–0.90	0.49*	0.46–0.53
2018	0.90*	0.85–0.95	0.81*	0.76–0.86
2019	0.90*	0.86–0.95	0.93*	0.87–0.99
2020	0.90*	0.85–0.95	0.95	0.89–1.02
2021	0.90*	0.86–0.96	1.03	0.97–1.10

Among the severe malaria incidences before and after SMC implementation, the fitted results have shown evidence of the impact of SMC each year during the first four rounds (2016 to 2019). However, starting from 2020, in contrast to the uncomplicated malaria cases, there has been no evidence of the impact of SMC in Cameroon. The first year registered a proportional reduction of 10% (IRR = 0.90, 95% CrI 0.84–0.97), followed by the greatest proportional reduction of 51% (IRR = 0.49, 95% CrI 0.46–0.53) in 2017. During the third round in 2018, a significant decrease of 19% in the impact of SMC on the incidence of severe malaria was observed, which further decreased in the fourth round in 2019, to 7% (IRR = 0.93, 95% CrI 0.87–0.99).

## Discussion

This study presents the initial assessment of the impact of SMC on malaria morbidity in Cameroon using routine malaria data. Throughout the entire implementation period (2016–2021), a consistent decrease in uncomplicated malaria incidence associated with SMC was observed. Additionally, a reduction in severe malaria incidence following SMC implementation during the first four rounds (2016–2019) was also observed. Except for 2017, when the reduction in the incidence of uncomplicated and severe malaria surpassed 15%, the measured impact of SMC on the incidence of uncomplicated malaria ranged between 10% and 12% for the other years. However, no reduction in the incidence of severe malaria has been reported since 2020. A similar decrease was observed in many other studies in Africa [4, 8, 11–16]. Using data from the Demographic Health Surveys, De Cola et al. [4] found strong evidence of a reduction in malaria prevalence measured by rapid diagnostic tests and microscopy separately in Burkina Faso and Nigeria. However, due to the age group of the Demographic and Health Survey population, their intervention group was 6–59 months, which excluded those between 3 and 5 months. A study by Kirakoya-Samadoulougou et al. [8] compared children under 5 years in an area that did not implement SMC with a group that implemented two rounds of SMC in Burkina Faso. The study observed a decrease in uncomplicated (31%) and severe (27%) malaria cases associated with the first two rounds of SMC. Furthermore, by utilizing routine data from DHIS2 and the same comparison group (individuals under 5 years) within a double-difference framework, the ACCESS SMC Project observed a reduction in malaria incidence among children under 5 years due to SMC in Burkina Faso and Gambia in 2015 and 2016. In separate studies conducted in Mali [12] and Burkina Faso [16] also, the authors reported a substantial decrease in malaria incidence owing to SMC implementation. Moreover, Konaté et al. [13] reported a significant reduction in malaria incidence in Dangassa village (Mali) after 2 years of implementation, both in 2015 and 2016, compared to 2013. Oyibo et al. [17] found a significant reduction in malaria parasite prevalence in 25 of 36 states with geographic or temporal variations in the reduction of malaria burden associated with SMC administration. However, our results contrast with those observed by Moundine et al. [18] in Chad, where an average increase of 1.58‰ in malaria incidence was associated with SMC implementation. This study was conducted in village/health district areas and used data from surveys conducted in these sampled villages/health districts.

This study has several strengths. The model used a population of children over 5 years from the same geographical area as the comparison group, which enabled us to account for the potential confounding factors, such as the potential decreasing effect of the 2016, 2019, and 2021 long-lasting insecticidal net campaigns, the impact of data quality, and the influence of health workers’ capacity building. Malaria incidence depends on certain non-observable variables such as entomological data, distance to a water body, and migration data which are not routinely collected during data collection [19]. Moreover, the study’s accuracy was enhanced by using monthly administrative data generated by health facilities over a long period (132 months). The inclusion of health programme data, consideration of malaria transmission seasonality, and analysis of spatial and temporal trends allowed for the generation of reproducible estimates of malaria incidence. The proposed method employed in this study successfully addressed missing data challenges and demonstrated its applicability to other routine data in Cameroon, allowing the reallocation of scarce resources to prioritized target areas and addressing financial limitations in the National Malaria Control Programme.

Despite the good agreement between the observed and modelled data, this study has several limitations. First, the data used were exclusively based on malaria cases from health facilities. Second, the population data used were not from the census but were estimated data from the National Institute of Statistics in collaboration with the Ministry of Public Health. The third limitation concerns the choice of the comparison group. In Cameroon, the SMC has been implemented directly in all health districts of the two eligible regions since the first round in 2016. This makes it difficult to choose a comparison group, especially a group of children under 5 years of age, in a non-SMC area with similar environmental conditions because the non-selected regions possess different climatic characteristics and are not eligible for SMC. In our study, some potentially important factors were assumed do not have a differential effect on malaria incidence among children under 5 years, compared to the comparison group during the SMC period. These include the use of insecticide-treated nets and the provision of access to malaria diagnosis and treatment. For example, in 2011 and 2014, the Government of Cameroon introduced a free-of-charge treatment for uncomplicated and severe malaria in children under 5 years. Additionally, efforts have been made to improve the availability of malaria treatment commodities in health facilities. These factors may have had different effects on the two age groups. Because information on net use was not available for the study period, especially for those aged over 60 months, no differential effect of long-lasting insecticidal net use on malaria incidence between the two groups was assumed. Therefore, the model used did not consider this variable for adjustment. Fourth, the eligible age to receive SMC is 3–59 months in Cameroon; individuals older than 60 months, who do not constitute a vulnerable group, may have a lower propensity to develop malaria during the period of high-transmission. This could have led to bias in the estimates of the impact of the SMC. Fifth, the incidence of malaria is often strongly correlated with climatic factors. In this study, climatic factors were assumed to affect both groups in a similar manner.

In these results, despite quantifying the effect of the SMC, the lack of a reduction in severe malaria incidence observed since 2020 raises the challenges of this intervention in reducing the malaria burden in Cameroon. Finally, this study did not account for the impact of the initial period of SMC implementation on the intervention. SMC was implemented in all health districts during the same months. Questions regarding seasonality and the beginning of the high-transmission period in eligible health districts should be the aim of further studies.

## Conclusion

Using routine malarial data from the HMIS in Cameroon, the effects of SMC were assessed. The fitted analysis revealed a robust association between the incidence of uncomplicated malaria and the SMC during all implementation periods in Cameroon (2016 to 2021). However, it was observed that only the first 4 years of SMC implementation (2016–2019) resulted in a significant decline in the incidence of severe malaria. For both uncomplicated and severe malaria incidence, the most substantial proportional reduction was observed in the second year of implementation (2017). This study supports the widespread adoption of the SMC strategy to alleviate the malaria burden in Cameroon as well as the value of utilizing routine malaria data for timely and efficient assessment of the effectiveness of the strategy.

### Supplementary Information

**Additional file 1.** Statistical modeling details.

**Additional file 2.** Metric measures for the accuracy of the clinical malaria prediction.

**Additional file 3.** Overall temporal marginal risk from full model.

**Additional file 4.** Posterior distribution of parameters.

## Data Availability

The datasets used and all code for data cleaning and analysis associated with the current submission are available from the corresponding author on reasonable request.

## Abbreviations

BCI: Bayesian Credible Interval
CAR: Conditional Autoregressive
CrI: Credible Interval
DHIS2: District Health Information System, version 2
HMIS: Health Monitoring Information System
IRR: Incidence Rate Ratio
SMC: Seasonal Malaria Chemoprevention
WHO: World Health Organization
