# Supplementary material for: A Bayesian spatio-temporal framework to assess the effect of seasonal malaria chemoprevention on children under 5 years in Cameroon from 2016 to 2021 using routine data
Source: Malar J. 2023 Nov 11;22:347. doi: 10.1186/s12936-023-04677-1 (PMC10640753; doi:10.1186/s12936-023-04677-1)
Supplement: Supplementary file 1 — Additional file 1. Statistical modeling details. [file 12936_2023_4677_MOESM1_ESM.docx]

## **Model Specification**

Three components characterize a Bayesian disease mapping model [1]: the data model (i.e., the distribution of the data given the parameters), the process model (i.e., the description of underlying spatial trends), and the parameter model (i.e., the prior distribution of the parameters to be estimated) [2].

### ***Data model***

The data model can be written as follows:

$Y_{it}\sim Negative Binomial \left( p_{it},\theta\right)$,

$p_{it}=\frac{\theta}{\theta+\mu_{it}}$,

where $Y_{it}$ is the number of uncomplicated (severe) malaria-confirmed cases reported in each health district $i (i=\left\{ 1, \ldots,45 \right\} represents a unique index for each heath district)$ at time $t (t=\{1, \ldots,132\}$ represents the time in months-year since the start of the study), $\theta$ is the dispersion parameter, and $\mu_{it}$ is the average monthly number of uncomplicated (severe) malaria-confirmed cases in district $i$.

$t=k+12*(j-1)$*,*

where $j = \left\{ 1, \ldots,11 \right\} is the number of year and k = \left\{ 1, \ldots,12 \right\} the number of month.$

### ***Process model***

To develop our process model, we use the segmented regression approach [3] which is widely used for the analysis of ITS data [4]. Let denote $T_{smc}^{k}$the time point (in months) since the start of the intervention during the $k-th$ year of implementation and $T_{withdrawal}^{k}$ the time point (in months) immediately following the end of the intervention during the $k-th$ year of implementation. The process is modeled through a log linear model that specifies the linear predictor $log(\mu_{it})$:

$\log\left( \mu_{it} \right)=\log\left( E_{it} \right)+\beta_{0}+{\beta_{1}G+ \beta}_{2}Gt+\sum_{k=1}^{6} \beta_{3k}T_{smc}^{k} X_{smc}^{k}+\sum_{k=1}^{6} \beta_{4k}T_{withdrawal}^{k} X_{withdrawal}^{k}+ \sum_{k=1}^{6} \beta_{5k}GT_{smc}^{k} X_{smc}^{k}+\sum_{k=1}^{6} \beta_{6k}G T_{withdrawal}^{k} X_{withdrawal}^{k}+{v_{i}+u_{i}+{\gamma_{t}+\varphi}_{t}+\delta_{it}+A}_{j}+S_{j}+\varepsilon_{it}$*,*

where $E_{it}$ is the offset population of health district $i (i=\left\{ 1, \ldots,45 \right\} represents a unique index for each heath district)$ at time $t: t$ represents the time in months-year since the start of the study ($t=\{1, \ldots,132\}$, $G$ represents the intervention group ($G=1$) or control group ($G=0$), , $k$ represents the year ($k=\{1, \ldots,6\}$) of implementation, $X_{smc}^{k}$is a dummy variable indicating the intervention period during the $k-th$ year of implementation and $X_{withdrawal}^{k}$ represents a dummy variable indicating the withdrawal period of intervention during the $k-th$ year of implementation.

$G=\left\{ \begin{aligned} 1, &For Intervention Group \\ 0, &For Comparison Group \end{aligned} \right.$*.*

$\beta_{0}$: represents the intercept at $t=0$.

$\beta_{1}$: represents the difference in intercept at $t=0$.

$\beta_{2}$: represents the slope difference between the intervention and control group in the pre-intervention period.

$\beta_{3k}$: represents the slope change following the intervention (using the interaction between time and intervention) during the $k-th$ year of implementation.

$\beta_{4k}$: represents the remaining slope change in the withdrawal period after the $k-th$ year of implementation.

$\beta_{5k}$: represents the impact of SMC during the $k-th$ year of implementation: the slope difference between the intervention and control group associated with the intervention during the $k-th$ year of implementation.

$\beta_{6k}$: represents the slope difference between the intervention and control group in the withdrawal period during the $k-th$ year of implementation.

In addition, $v_{i}$ + $u_{i}$ and $\gamma_{t}+\varphi_{t}$ are, respectively, the spatial and temporal random effect of district $i (i=\left\{ 1, \ldots,45 \right\})$ at time $t (t=\left\{ 1, \ldots,132 \right\})$: $v_{i}$ is the spatially unstructured random effect modeled as exchangeable, $u_{i}$ another area-specific effect, which we now model as a spatially structured random effect; $\gamma_{t}$ represents the temporally structured effect; $\varphi_{t}$represents the temporally unstructured effect; and $\delta_{it}$represents the space-time interaction term.

Finally, we included an annual trend ($A_{j}$) and annual seasonal ($S_{j}$) random effects for each year $j = \{1, \ldots,11\}$ to account for inter-annual variance, and $\varepsilon_{it}$ captures supplementary variability in the data not described by other model components.

### ***Parameters model***

Prior distributions were assigned for all model parameters.

In our study, we used the Conditional Autoregressive (CAR) model [5] to specify the spatially structured area-specific effect $u$ = {$u_{1}$, …,$u_{n_{\delta_{i}}}$}. So, each $u_{i}$ conditional on the neighbor $u_{-i}$ follows a normal distribution with mean equal to the average of neighboring districts $u_{k}$ _,_$k\in\delta_{i}$ and variance inversely proportional to the number of neighbor districts $n_{\delta_{i}}$, that is:

$u_{i}/u_{-i} \sim Normal ( \mu_{i}+ \sum_{j=1}^{n} r_{ij}\left( u_{j}-\mu_{j} \right), \tau_{i}^{2})$,

$\tau_{i}^{2}=\frac{\sigma_{u}^{2}}{n_{\delta_{i}}}$,

with $u_{i}$ as the spatially structured random effect for health district $i. \delta_{i}$ and $n_{\delta_{i}}$ respectively, represent the set of neighbors and the number of neighbors for a specific health district $i$; $\mu_{i}$ is the average of the neighboring districts; $u_{k}$, $k \epsilon\delta_{i}$; $\tau_{i}^{2}$ is a spatial variance inversely proportional to the number of neighboring districts $n_{\delta_{i}}$; and $r_{ij}$ indicates the spatial proximity defined as:

$$r_{ij}=\frac{a_{ij}}{n_{\delta_{i}}},$$

$a_{ij}=\left\{ \begin{aligned} 1, &i \neq j and\mathrm{areas}i\mathrm{and}j are neighbours \\ 0, &otherwise \end{aligned} \right.$.

Considering $R$as the neighborhood matrix, $R$ is then defined as the matrix of generic element $r_{ij}$, assuming that $R$is a 45x45 matrix.

Therefore, we have:

$$u_{i}/u_{-i}\sim Normal \left( \mu_{i}+ \frac{1}{n_{\delta_{i}}} \sum_{j=1}^{n} a_{ij}\left( u_{j}-\mu_{j} \right), \tau_{i}^{2} \right).$$

The spatially unstructured component $v_{i}$ is modeled using a Gaussian process:

$v_{i} \sim N ( 0 , \sigma_{v}^{2})$.

The temporally structured effect $\gamma_{t}$ is modeled dynamically using an autoregressive process order 2 (AR2) and defined as follows:

$\gamma_{t}/\gamma_{t-1}/\gamma_{t-2} \sim Normal ( \gamma_{t-1}+\gamma_{t-2} , \sigma_{\gamma}^{2})$.

The temporally unstructured effect $\varphi_{t}$ is using a Gaussian process:

$\varphi_{t} \sim N ( 0 , \sigma_{\varphi}^{2})$.

The annual trend ($A_{j}$) is modeled through an autoregressive process order 1 (AR1) and defined as follows:

$A_{j}/A_{j-1} \sim Normal ( A_{j-1} , \sigma_{A}^{2})$.

The seasonal pattern ($S_{j}$) was captured with a cycle of *p =*$12$ months. The vector S = {$S_{1}$, …,$S_{n=132}\}$, *n* ˃ *p* is obtained assuming that $\sum_{j=t}^{t+p-1} S_{j}$ are independent Gaussian with precision $\sigma_{S}^{2}$.

A non-informative normal prior distribution was assumed for the regression coefficients $\beta_{1}, \beta_{2}, \beta_{3k},\beta_{4k}, \beta_{5k},\beta_{6k}$ ($k$ = {1, …,6}): a Gamma distribution with mean 1 and variance 100.

$\beta_{1}, \beta_{2}, \beta_{3k}, \beta_{4k}, \beta_{5k},\beta_{6k} (k=\{1, \ldots,6\}) \sim Gamma (1, 100)$.

$\mathrm{Log}\left( \sigma_{u}^{2} \right) and Log(\sigma_{v}^{2})$ are modeled as minimally informative prior:

$${Log(\sigma}_{u}^{2}), {Log(\sigma}_{v}^{2}) \sim LogGamma \left( 1, 0.0001 \right).$$

$\sigma_{\gamma}^{2}, \sigma_{\varphi}^{2}, \sigma_{A}^{2}, \sigma_{S}^{2}$ are modeled as a non-informative Gaussian prior distribution:

$$\sigma_{\gamma}^{2}, \sigma_{\varphi}^{2}, \sigma_{A}^{2}, \sigma_{S}^{2} \sim Gaussian \left( 1, 0.0001 \right).$$

### ***Estimation method***

To perform this model, we used Integrated Nested Laplace Approximation because it takes much less time than estimation using Markov Chain Monte Carlo methods [6]. The INLA method computes the integrals of the high-dimensional latent components using the Laplace approximation device. This laplace approximation, combined with efficient algorithms and numerical tricks and approximations, ensures that the INLA approach approximates quickly but accurately the posterior marginal densities of the parameters of interest [7]. Descriptive statistics were performed in Stata (version 14), and the controlled interrupted times series regression analyses were performed using R software, version 4.2.2 (R Foundation for Statistical Computing, Vienna, Austria).

For each outcome (uncomplicated and severe malaria incidence), 07 models were fitted to capture the (potential) non-linearity in the malaria-dynamics. These models are: i) a model with spatial effect and time as fixed effect, ii) a model without spatial effect and with time as fixed effect, iii) a model without spatial effect and with time as random effect (AR2) and iv) a model with spatial effect, time as random effect (AR2) and with space-time interaction type I, II, III and IV. The best model was obtained based on the lowest Deviance Information Criterion (DIC) value, which suggested a model with spatial and time as random effects and interaction space-time type II, which combines the structured temporal main effect $\gamma_{t}$ and the unstructured spatial effect $v_{i},$ as the best model, compared to other models fitted, shown in Table 1. For instance, the DIC was 141,553 for model on uncomplicated malaria incidence on children under five years with spatial as a random variable coupled with time as a fixed effect.

| Models | DIC | |
| --- | --- | --- |
|  | **Uncomplicated malaria cases** | **Severe malaria cases** |
| Spatial with time as fixed effect | 141,533 | 138,237 |
| No spatial with time as fixed effect | 146,551 | 142,496 |
| No spatial with time as random effect (AR2) | 146,505 | 142,408 |
| Spatial with time as random effect (AR2) Type I | 139,308 | 138,007 |
| Spatial with time as random effect (AR2) Type II | 134,959 | 134,004 |
| Spatial with time as random effect (AR2) Type III | 141,501 | 138,157 |
| Spatial with time as random effect (AR2) Type IV | 135,356 | 139,233 |

**Table 1.** Comparison of models based on DIC

### ***Accuracy of the clinical malaria case prediction***

We used three metrics to assess the accuracy of our model to predict clinical malaria cases: Spearman correlation coefficient, R^2^, and Root Squared Mean Error (RMSE). As shown in Table 2, we can affirm that our model accurately predicted monthly malaria-confirmed cases in Cameroon.

| Metrics | Uncomplicated malaria cases (value) | Severe malaria cases (value) |
| --- | --- | --- |
| Spearman correlation coefficient | 0.994 | 0.993 |
| R^2^ | 0.989 | 0.991 |
| RMSE | 0.952 | 0.485 |

**Table 2.** Metric measures for the accuracy of our model

1. Semakula M, Niragire F, Faes C. Bayesian spatio-temporal modeling of malaria risk in Rwanda. PLoS ONE. 2020;15(9): e0238504. <https://doi.org/10.1371/journal.pone.0238504>.
2. Lesaffre E, Lawson AB. Bayesian biostatistics. Statistics in practice. United Kingdom: John Wiley & Sons; 2012. <https://doi.org/10.1002/9781119942412>.
3. Habib N, Steyn PS, Boydell V, Cordero JP, Nguyen MH, Thwin SS, Nai D, Shamba D, Kiarie J; CaPSAI Project Team. The use of segmented regression for evaluation of an interrupted time series study involving complex intervention: the CaPSAI project experience. Health Serv Outcomes Res Methodol. 2021;21(2):188-205. doi: 10.1007/s10742-020-00221-9. Epub 2020 Nov 24. PMID: 34720688; PMCID: PMC8550724.
4. Wagner AK, Soumerai SB, Zhang F, Ross-Degnan D. Segmented regression analysis of interrupted time series studies in medication use research. J Clin Pharm Ther. 2002 Aug;27(4):299-309. doi: 10.1046/j.1365-2710.2002.00430.x. PMID: 12174032.
5. Besag J, York J, Mollie A. Bayesian image restoration with two applications in spatial statistics. Ann Inst Statist Math. 1991;43:1. <https://doi.org/10.1007/BF00116466>.
6. Carroll R et al. Comparing INLA and OpenBUGS for hierarchical Poisson modeling in disease mapping. Spatial and Spatio-temporal Epidemiology. 2015;14-15:45–54. https://doi.org/10.1016/j.sste.2015.08.001. PMID: 26530822.
7. Thomas Opitz. Latent Gaussian modeling and INLA: A review with focus on space-time applications. 2016. hal-01394974
