# Supplementary material for: A Bayesian spatio-temporal framework to assess the effect of seasonal malaria chemoprevention on children under 5 years in Cameroon from 2016 to 2021 using routine data
Source: Malar J. 2023 Nov 11;22:347. doi: 10.1186/s12936-023-04677-1 (PMC10640753; doi:10.1186/s12936-023-04677-1)
Supplement: Supplementary file 2 — Additional file 2. Metric measures for the accuracy of the clinical malaria prediction. [file 12936_2023_4677_MOESM2_ESM.docx]

**Additional file 2:**


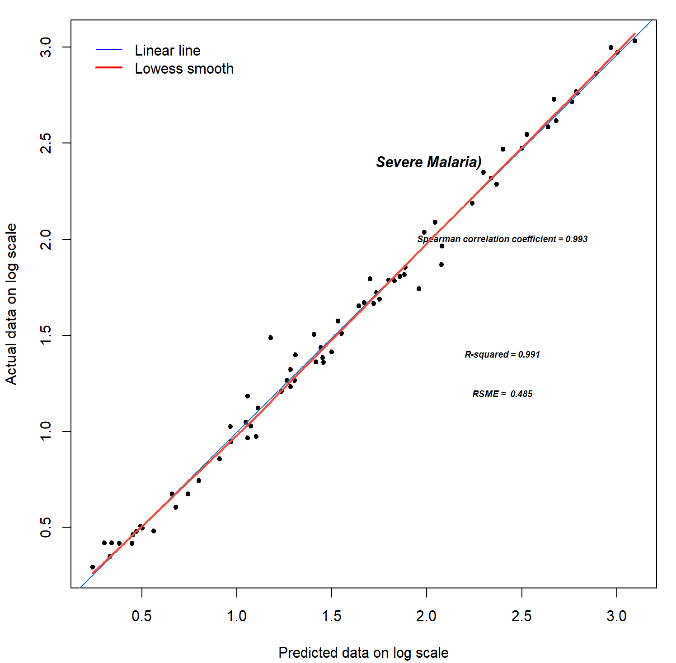

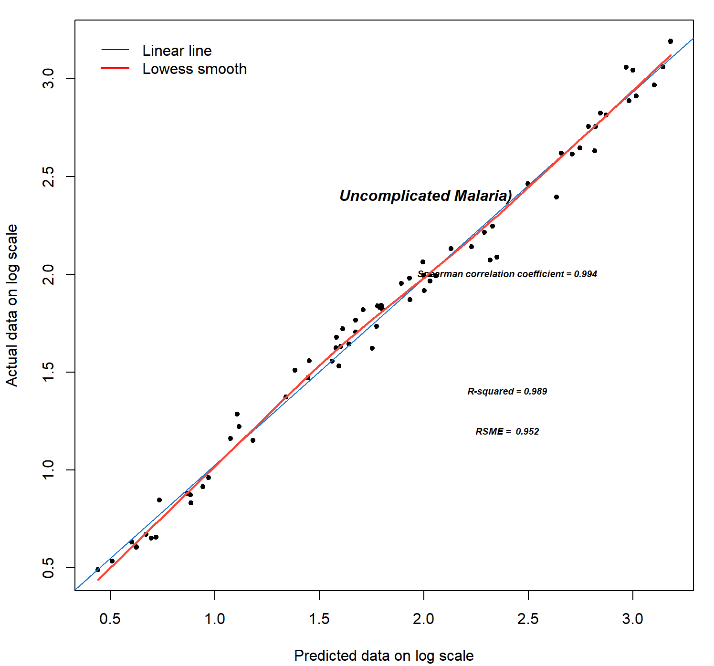


**Figure S1.** Metric measures for the accuracy of the clinical malaria prediction. **(Left)** Linear and smooth relationship between the actual observed data collected during the last three years (2019 and 2021) and predicted values of uncomplicated malaria cases during the same period. **(Right)** Linear and smooth relationship between the actual observed data collected during the last three years (2019 and 2021) and predicted values of severe malaria cases during the same period
