# Supplementary material for: A Bayesian spatio-temporal framework to assess the effect of seasonal malaria chemoprevention on children under 5 years in Cameroon from 2016 to 2021 using routine data
Source: Malar J. 2023 Nov 11;22:347. doi: 10.1186/s12936-023-04677-1 (PMC10640753; doi:10.1186/s12936-023-04677-1)
Supplement: Supplementary file 3 — Additional file 3. Overall temporal marginal risk from full model. [file 12936_2023_4677_MOESM3_ESM.docx]

**Additional file 3:**


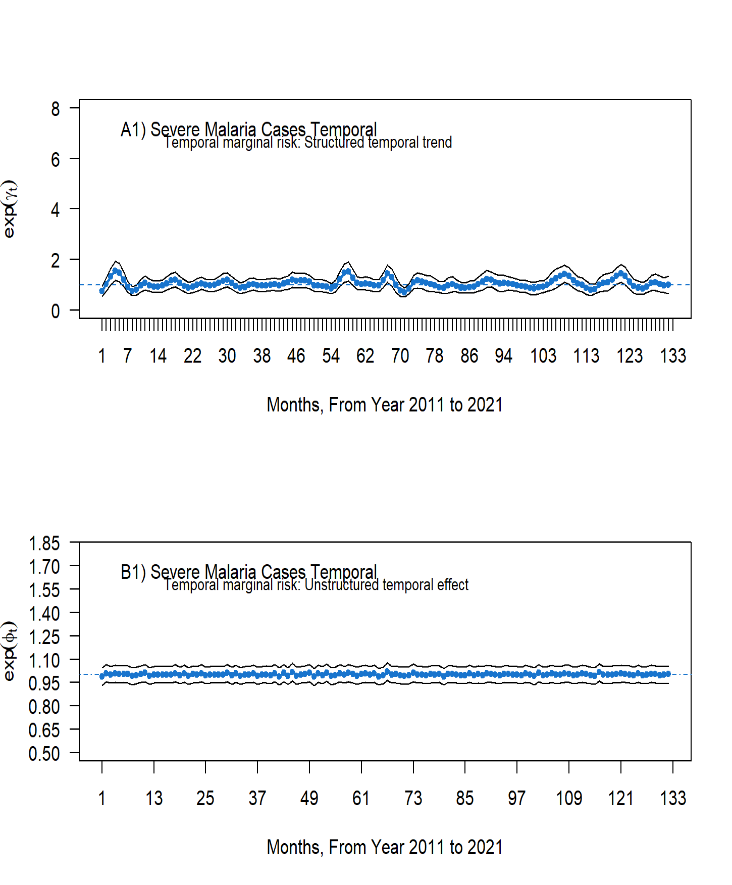

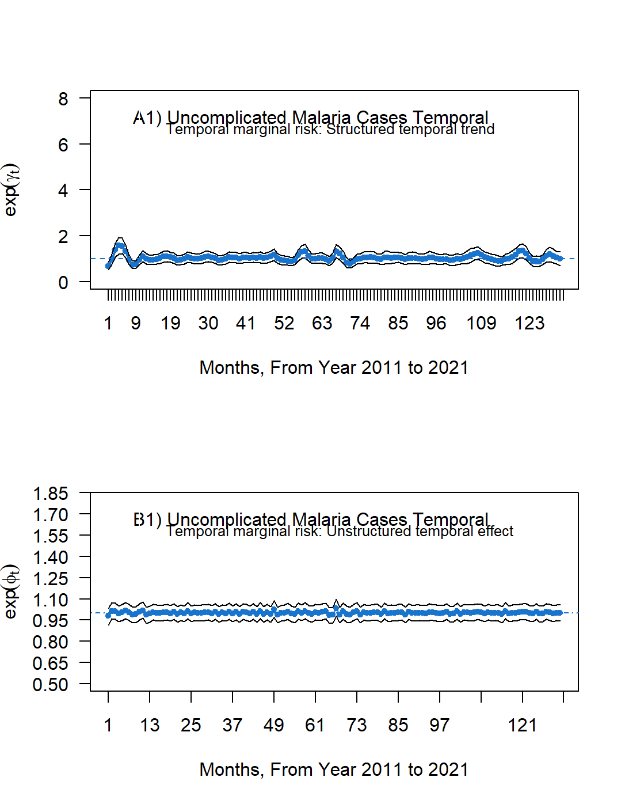


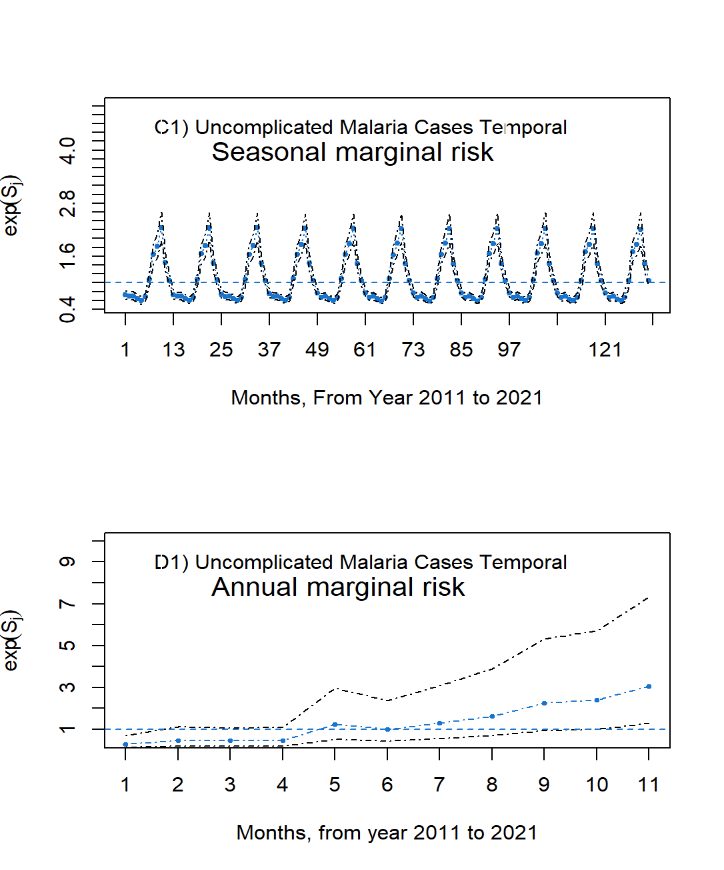

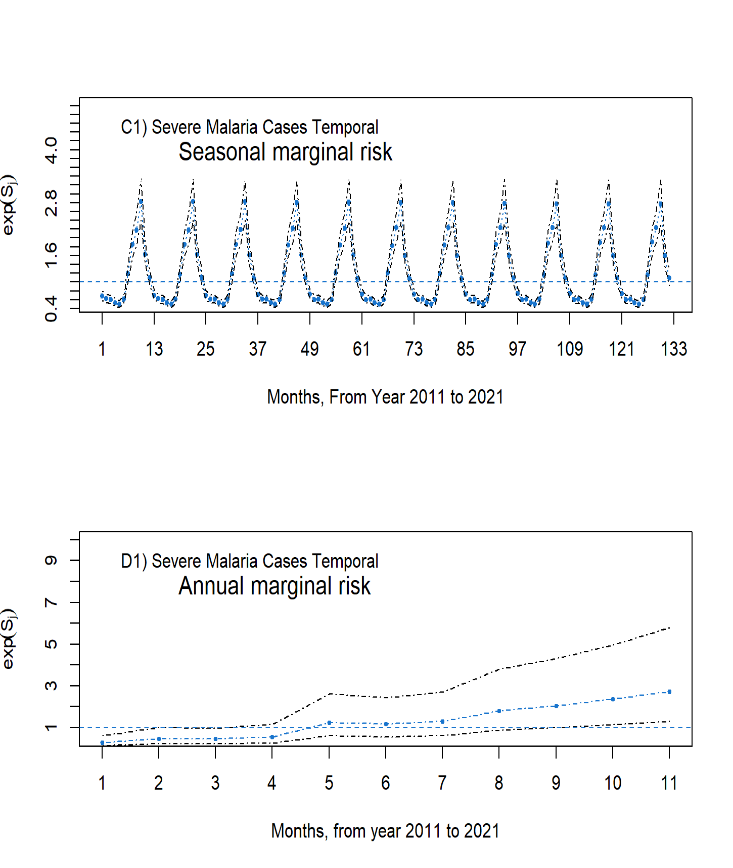


**Figure S2**. Overall temporal marginal risk from full model for uncomplicated (left) and severe (right) malaria incidence in the North and Far North regions in Cameroon with 95% CrI: A) Monthly temporally structured trend 𝑒𝑥𝑝(𝛾𝑡), B) Monthly temporally unstructured trend 𝑒𝑥𝑝(𝜙𝑡), C) Seasonal effect 𝑒𝑥𝑝(𝑆𝑗), and D) Annual temporally unstructured trend 𝑒𝑥𝑝(𝑇𝑗)
