# Supplementary material for: A Bayesian spatio-temporal framework to assess the effect of seasonal malaria chemoprevention on children under 5 years in Cameroon from 2016 to 2021 using routine data
Source: Malar J. 2023 Nov 11;22:347. doi: 10.1186/s12936-023-04677-1 (PMC10640753; doi:10.1186/s12936-023-04677-1)
Supplement: Supplementary file 4 — Additional file 4. Posterior distribution of parameters. [file 12936_2023_4677_MOESM4_ESM.docx]

**Additional file 4:**


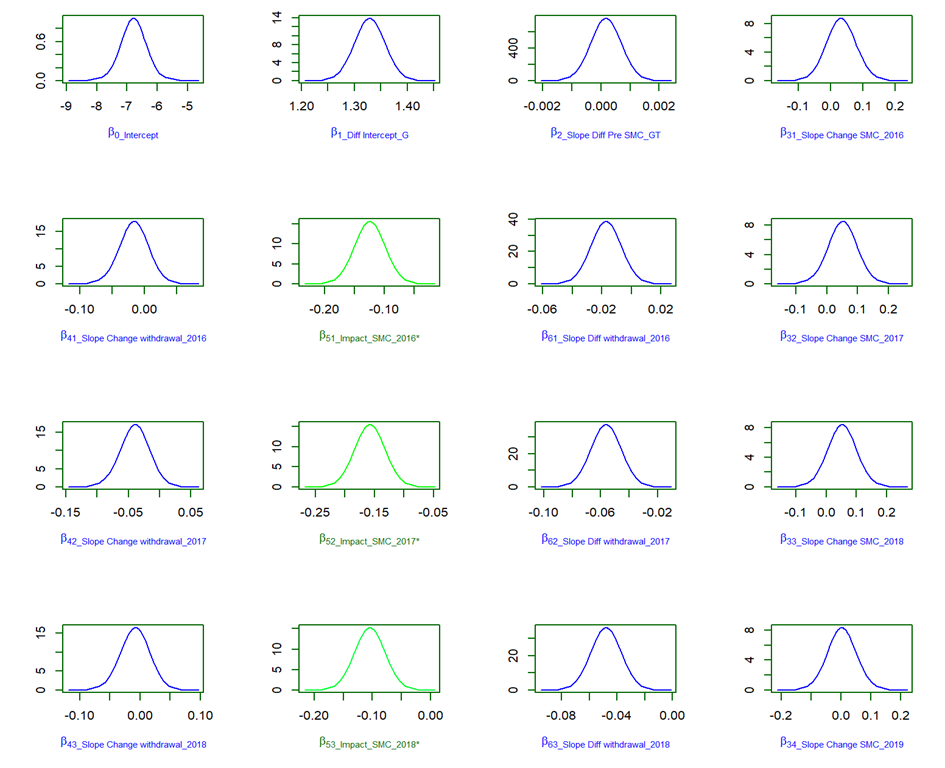


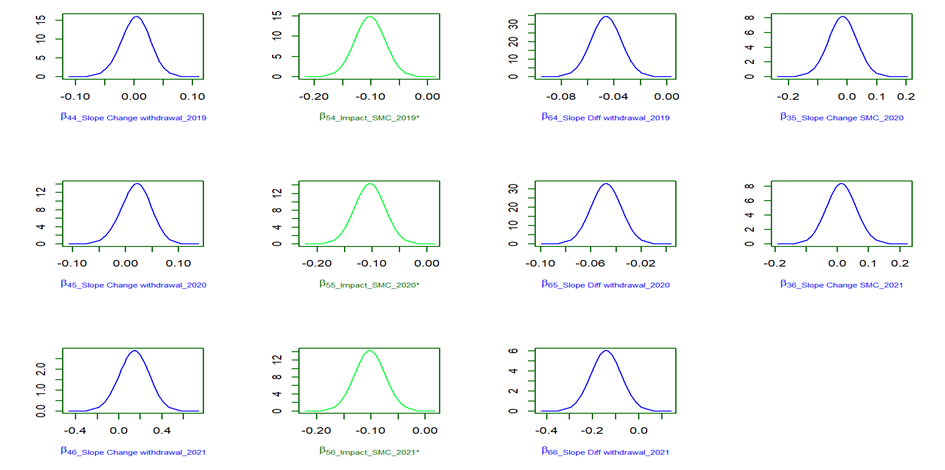


**Figure S3**. Posterior Distribution of parameters for the Bayesian spatial model assessing impact of the SMC on the uncomplicated malaria incidence among children under 5 years aged in the North and Far North regions of Cameroon. *Denotes significant impact


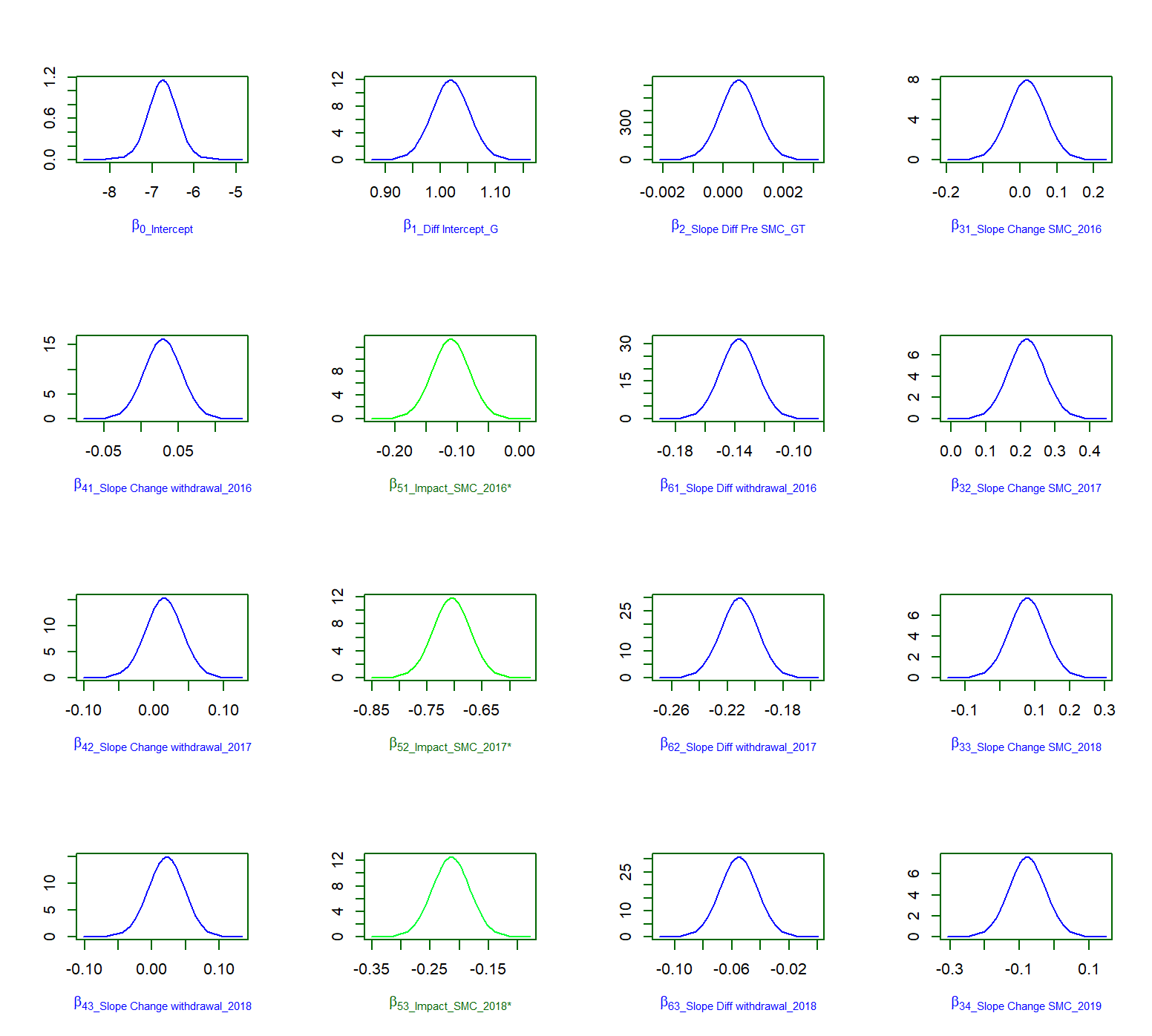


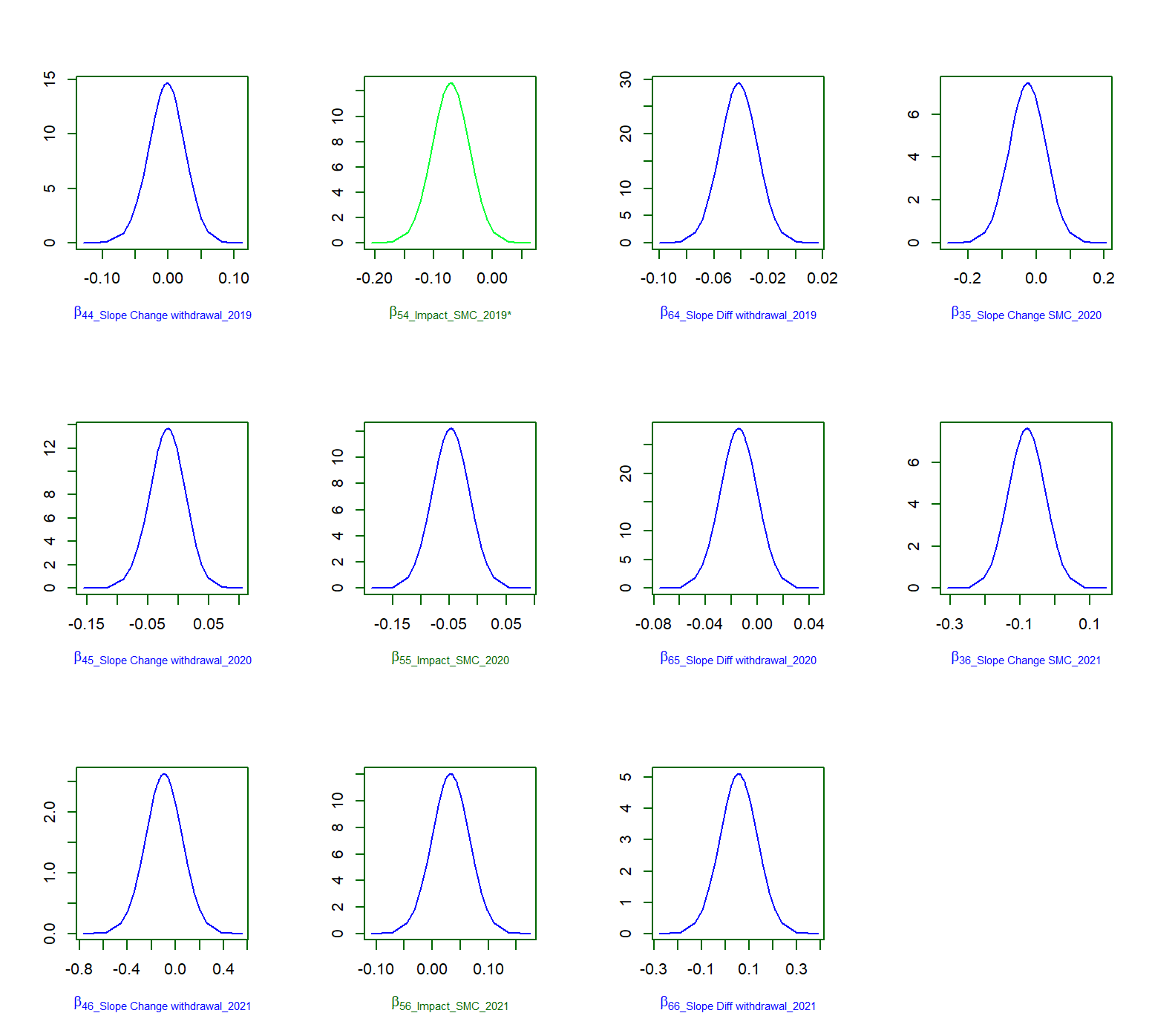


**Figure S4**. Posterior Distribution of parameters for the Bayesian spatial model assessing impact of the SMC on the severe malaria incidence among children under 5 years aged in the North and Far North regions of Cameroon. *Denotes significant impact
